# The important role of connexin 43 in subarachnoid hemorrhage-induced cerebral vasospasm

**DOI:** 10.1186/s12967-019-02190-1

**Published:** 2019-12-30

**Authors:** Le Yang, Jian Yan, Jin-An Zhang, Xin-Hui Zhou, Chao Fang, Er-Ming Zeng, Bin Tang, Jian Duan, Guo-Hui Lu, Tao Hong

**Affiliations:** Department of Neurosurgery, The First Affiliated Hospital of Nanchang University, 17 Yong Wai Zheng Street, Nanchang, China

**Keywords:** Subarachnoid hemorrhage, Cerebral vasospasm, Gap junction, Connexin 43, Protein kinase C

## Abstract

**Background:**

Gap junctions are involved in the development of cerebral vasospasm (CVS) after subarachnoid hemorrhage (SAH). However, the specific roles and regulatory functions of related connexin isoforms remain unknown. The aim of this study was to investigate the importance of connexin 43 (Cx43) in CVS and determine whether Cx43 alterations are modulated via the protein kinase C (PKC) signaling transduction pathway.

**Methods:**

Oxyhemoglobin (OxyHb)-induced smooth muscle cells of basilar arterial and second-injection model in rat were used as CVS models in vitro and in vivo. In addition, dye transfer assays were used for gap junction-mediated intercellular communication (GJIC) observation in vitro and delayed cerebral ischemia (DCI) was observed in vivo by perfusion-weighted imaging (PWI) and intravital fluorescence microscopy.

**Results:**

Increase in Cx43 mediated the development of SAH-induced CVS was found in both in vitro and in vivo CVS models. Enhanced GJIC was observed in vitro CVS model, this effect and increased Cx43 were reversed by preincubation with specific PKC inhibitors (chelerythrine or GF 109203X). DCI was observed in vivo on day 7 after SAH. However, DCI was attenuated by pretreatment with Cx43 siRNA or PKC inhibitors, and the increased Cx43 expression in vivo was also reversed by Cx43 siRNA or PKC inhibitors.

**Conclusions:**

These data provide strong evidence that Cx43 plays an important role in CVS and indicate that changes in Cx43 expression may be mediated by the PKC pathway. The current findings suggest that Cx43 and the PKC pathway are novel targets for developing treatments for SAH-induced CVS.

## Introduction

CVS is thought to be a severe complication of SAH. However, the pathogenesis of CVS is not completely understood, and no definitive treatment has been established. Once aneurysm rupture occurred, blood pours into the subarachnoid space even to the brain parenchyma and ventricles. The intracranial pressure rises sharply and might increase enough to affect cerebral perfusion and cause global ischemia. Due to CVS, maximal 7–10 days after onset of SAH, the presence of blood in the subarachnoid spaces triggered and associated with DCI, persistent neurological deficits and long-term neurological disability. DCI is related to the development of CVS, as it is the most important adverse prognostic factor of outcome and a major cause of morbidity and mortality in SAH patients [1]. The pathogenesis of DCI is hypothesized to be multifactorial, including angiographic vasospasm, ischemia, microthrombosis and microcirculation constriction [2–4]. Due to DCI is among the most important adverse prognostic factors for outcome after SAH [4], it’s of great necessity to explore new targets dealing with the progress of pathology in DCI based on previous SAH animal models [5–7]. In our previous study [8], we supported the hypothesis that gap junction blockers may relieve the CVS after SAH via cerebral angiography and morphologic study, suggested that gap junctions may play an important role in the pathogenesis of CVS.

Gap junction channels are formed by members of a family of proteins known as connexins [9]. Among them, Cx43 is the most abundant and the major connexin in vascular smooth muscle [10]. Liao Y et al. [11] reported that the conditional knockout of Cx43 in vascular endothelial cells resulted in hypotension, indicating that altered Cx43 expression may be related to irregular vasomotion. In our previous study [8, 12], we reported that Cx43 levels were higher and that vasospasm occurred in the BAs at 7 days after SAH in animals. But, the specific role of Cx43 in DCI after SAH is not well understood.

SAH-induced CVS most often occurs between day 3 and day 7 [13–15], indicating that it may be associated with one or more byproducts of blood breakdown in the subarachnoid space, where the exterior surfaces of the cerebral vessels are exposed to blood and byproducts of its breakdown. The breakdown of blood results in the release of OxyHb-derived free radicals, which lead to inhibition of ATP-dependent calcium pumps [16, 17], alterations in vasomotor tone including the release of vasoactive eicosanoids and endothelin from the vessel wall, inhibition of endothelium-dependent relaxation, scavenging of NO and possible influences on the development of CVS [18]. OxyHb-mediated CVS is dependent on the activation of PKC enzymes [19], which are broadly involved in vital cellular functions, including smooth muscle contraction [20].

Several studies have revealed that in vasospastic cerebral arteries, PKC activity is significantly enhanced in the membrane fraction [21], and the time courses of the progression of CVS and PKC activation are well correlated, as revealed by an enzyme immunoassay study [22]. However, the specific mechanism of PKC activation leading to CVS is unclear. Joshi CN et al. [23] demonstrated an important role of Cx43 in the proliferation of vascular SMCs: increased Cx43 expression was significantly reversed in the presence of PKA, PKG and PKC inhibitor in thoracic aorta SMCs. In addition, S Nishizawa et al. proposed that PKC isoforms potentially play a role in the initiation and maintenance of delayed CVS [24] implicating the PKC pathway in Cx43 expression and Cx43-mediated GJIC. But, to date, there is no information available regarding the possible involvement of PKC in Cx43 alterations and gap junction remodeling on cerebrovascular SMCs under spastic conditions. The purpose of the present study was to determine the actual role of Cx43 on DCI after SAH and its involvement in the PKC pathway to lay the groundwork for developing therapeutic strategies for SAH.

In the current study, the time-course changes in Cx43 both in vitro and in vivo were explored. The exact location of Cx43 expression in BA walls was detected using a laser-scanning confocal microscope. By specifically knocking down Cx43 in BAs using siRNA interference, we correlated the important role of Cx43 with DCI in a common SAH model. In addition, by designing an experimental therapeutic study using specific PKC inhibitors, we provide evidence that Cx43-mediated GJIC enhancement and DCI may be modulated via the PKC pathway, which could be used as reference data for developing and analyzing neuroprotective strategies in further studies.

## Materials and methods

### Rat cell culture and SAH model established in vitro

Rat BASMCs were isolated by enzymatic digestion as previously described [25]. As a major component of blood, OxyHb has been proven to be the principal cause of CVS and delayed neurological deficits following aneurismal SAH. It has also been used to induce the SAH model in vitro as described previously [26]. To mimic SAH in vitro, SMCs were exposed to OxyHb at 10^−6^ M with complete culture medium in all dishes. SMCs passaged for 3 generations were cultured to confluence. The total medium was then discarded. After rinsing twice with sterile PBS, complete culture medium with OxyHb was added. The dishes were then separated into four groups incubated with OxyHb for different times: 24 h in Group I, 48 h in Group II, 72 h in Group III, and 96 h in Group IV. To study the effects of PKC inhibition, Group II was further divided into three subgroups corresponding to a OxyHb-only group and OxyHb groups pretreated with two specific PKC inhibitors: (i) chelerythrine (CHE) [27] and (ii) GF 109203X (GF) [28]. The total protein of all the cells were collected and stored at − 80 °C until analysis of Cx43 via Western blot. All animal procedures were carried out in accordance with the Guide for the Care and Use of Laboratory Animals published by the US National Institutes of Health (NIH publication No. 86-23, revised 1985); animal use and welfare followed a protocol reviewed and approved by the First Affiliated Hospital of Nanchang University.

### Dye transfer assays

The intercellular dye transfer assay of Lucifer yellow (LY) (5% w/v in 150 mM LiCl) was evaluated by microinjecting the dye into a single BASMC and evaluating the diffusion of the dye into neighboring cells in a cell monolayer, as previously described [29]. Single cells were microinjected using a Picoliter Injector (model PLI-90, Harvard Apparatus, South Natick, MA, USA) containing the dye until the impaled cell was brightly fluorescent. 5 min. after injection, the cells were photographed by epifluorescence and the extent of intercellular transfer of LY was determined by counting the number of adjacent cells containing the dye tracer [30]. Experiments were repeated in triplicate per experiment.

### Induction of experimental SAH and experimental therapeutic study

Adult male Sprague–Dawley rats were provided by the Department of Laboratory Animal Science, Medical College of Nanchang University, Nanchang, China. The double-hemorrhage model of SAH used here was described previously [31]. Briefly, on both day 1 and 2, the rats were anesthetized using 3% isoflurane in an oxygen and nitrous oxide mixture (1:2). After the loss of the righting reflex, the rat was transferred to a heating blanket (Harvard Apparatus Ltd., UK) coupled to a rectal probe for the maintenance of body temperature (37.5 ± 0.5 °C). After a small incision in the atlanto-occipital membrane, 0.1 ml cerebrospinal fluid was withdrawn, and 0.2 ml non-heparinized blood was injected through the needle inserted into the cisterna magna. The head of the rat was placed in a nose-downward position for 20 min to distribute the blood into subarachnoid spaces after second injection. In a control “sham” group, the atlanto-occipital membrane was exposed but equivalent normal saline was injected to determine if the surgery significantly altered the outcome of the current study. The animals were euthanized by overdose with carbon dioxide (CO_2_) gas 1, 3, 5, 7 and 14 days after SAH induction, and the BAs were removed for Western blotting or immunohistochemistry.

Knockdown of Cx43 was performed by using an HVJ envelope (HVJ-E) transfection kit (GenomONE™, Cosmo Bio Co., Ltd., Tokyo, Japan), which is widely used for in vivo siRNA transfer [32, 33]. Cx43 siRNA (siGENOME SMARTpool, mouse GJA1, Thermo Scientific) [34, 35] was incorporated into the HVJ-E according to the manufacturer’s instructions. Two days prior to SAH induction, siRNA (5 pg in 10 μl sterile saline) was slowly injected into the cisterna magna as pretreatment, similar to the induction of SAH described above. siGENOME Non-Targeting siRNA Control Pool #2 (Thermo Scientific) was used as a negative control. In addition, the pharmacological effects of the two PKC inhibitors were examined in separate groups. Two days prior to SAH induction, 3 ml sterile PBS containing 5 μM CHE or GF was injected into the cisterna magna, and on day 3 and day 5 after SAH induction, we injected the same amount of each drug into the cisterna magna. Seven days after SAH induction, animals were reanesthesized to perform MR PWI or intravital fluorescence microscopy. After completion of these examinations, the BAs were removed for Western blotting or immunohistochemistry.

### MRI protocol and intravital fluorescence microscopy of rats

After SAH induction, the rats were randomly divided in two groups, which were respectively examined by functional MRI and intravital fluorescence microscopy on day 7, the day of the maximum CVS and DCI, as previous reported [36] with maximum vasospasm detected after day 7 using transcranial Doppler ultrasound. Each group was further divided into six subgroups as follows: (I) a sham-operated group, (II) an SAH-only group, (III) an SAH + Cx43siRNA group, (IV) an SAH + control siRNA group, (V) an SAH + CHE group and (VI) an SAH + GF group. For postanesthetic animals, MR PWI experiments were carried out using a 7.0T/30-cm imaging system (Bruker Biospec, Germany) with a gradient inset (121 mm internal diameter, 400 mT/m). For bolus tracking PWI of axial slices, a magnetic resonance gradient echo sequence was used near the position of hippocampus. After standardized intravenous injection of 0.2 ml Gd-DTPA (0.1 mmol/kg) at a flow rate of 0.15 ml/s, 22 sequential images were obtained in approximately 60 s. PWI post-processing was implemented with Signal Processing In Nmri software (SPIN, MRI Institute for Biomedical Research, Detroit, MI, USA). Relative regional (rr) blood flow (BF) was calculated semiquantitatively in different ROIs. In each animal, ROIs were selected in the hippocampus and the masseter muscle. The ratio of rr CBF to rr masseter muscle BF was defined to determine the reduction of CBF due to CVS [37]. For each group, a mean value of the ratio was calculated. In another group, the animals underwent an intravital fluorescence microscopy experiment.

For intravital fluorescence microscopy, each subgroup was assessed with intravital microscopy according to a previous study [38–40]. The methods of Friedrich B, et al. [38] with slight modification were used for microvessel assessment. Briefly, vessel diameters were quantified in the images captured by video and analyzed using IC Viewer software (Mauna Kea Technologies, Paris, France). The percentage of spastic vessels among all vessels in each group were calculated. Individual vessel constrictions were calculated by dividing the diameter of the most constricted vessel segments (Fig. 5a, white arrow) by the diameter of the nearest non-constricted vessel segment among each group. To confirm that non-constricted vessel segments indeed represent the baseline diameter of the vessel, microvessels were categorized by their degree of branching from the MCA (A1 to A6). The mean diameter of each vessel category was calculated and compared between sham-operated rats and SAH rats. No difference was detected between the groups, indicating that the non-constricted vessel segments in the rats subjected to SAH retained physiological baseline characteristics.

### Western blot analysis

Western blot analysis was used to analyze changes in the expression of Cx43 protein. Briefly, proteins from each group of BAs or SMCs were subjected to SDS-PAGE and then transferred to nitrocellulose membranes, which were blocked for 2 h in Tris-buffered saline Tween (TBST) containing 5% milk powder at room temperature. The anti-Cx43 antibody (MAB3067; Sigma, diluted 1:1000) and anti-GAPDH antibody (AB2302; Sigma, diluted 1:1000) were used to the membrane in saturation buffer. After extensively washing 5 times in TBST for 10 min each, the membranes were probed for 2 h with horseradish peroxide-conjugated goat anti-rabbit secondary antibody (diluted 1:2000 in saturation buffer). The durations of exposure to hyperfilm and incubation in ECL detection reagents were maintained consistent for all conditions. The intensity of the bands after Western blotting was determined by laser scanning of the films, followed by quantitative densitometric analysis using Kodak Digital Science 1D 2.0 Image software, and the protein expression levels of Cx43 were normalized to GAPDH.

### Statistical analysis

Data are presented as the mean ± SEM. Statistical significance of differences were assessed by one-way analysis of variance (ANOVA) followed by Student’s t-tests using IBM SPSS Statistics, Version 21. The results were considered significant for p < 0.05.

## Results

### In vitro detection of Cx43 protein levels in the experimental vasospasm model among cultured BASMCs and the involvement of the PKC pathway

**Fig. 1:**
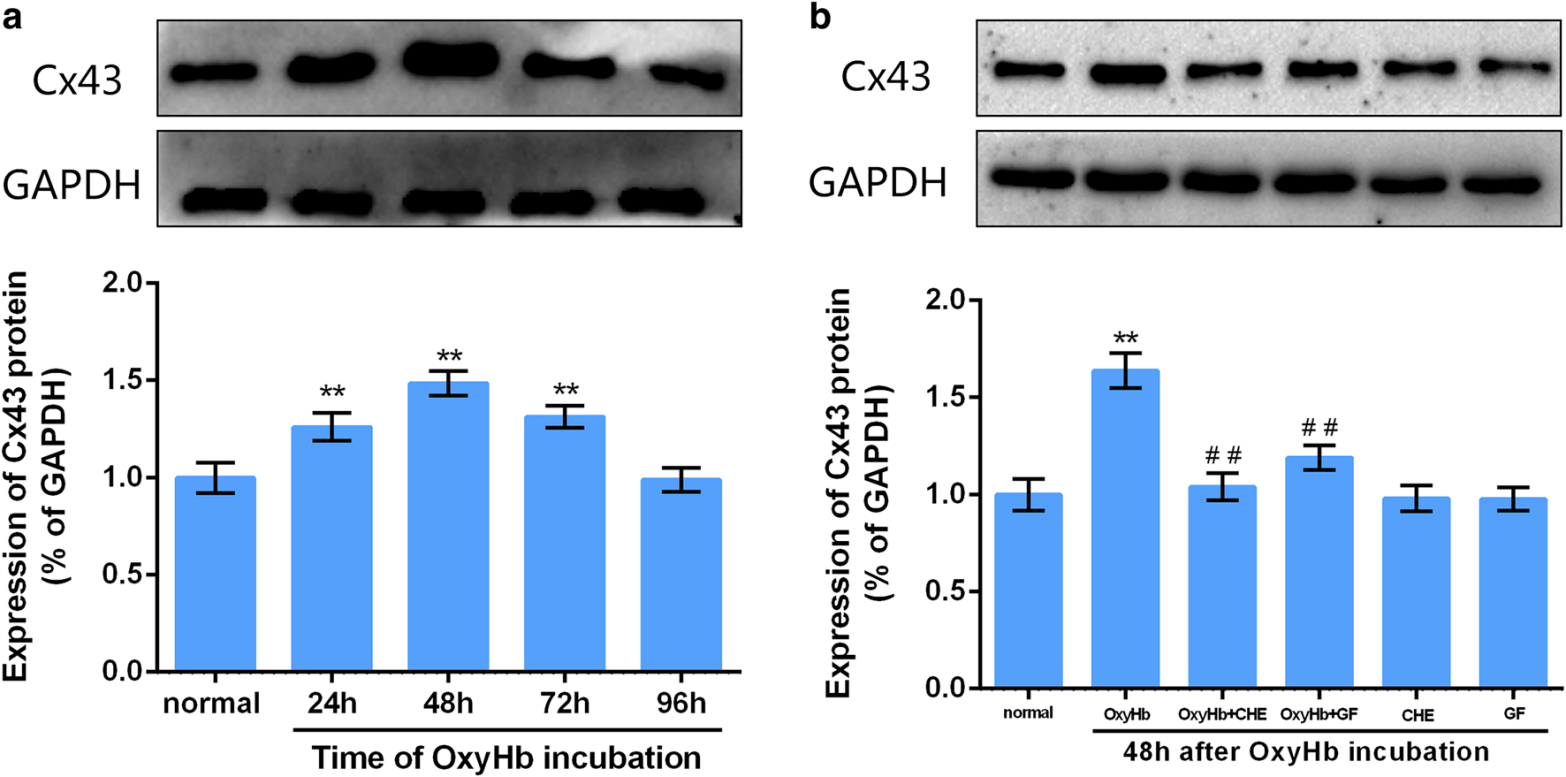
Time course of the effect of OxyHb on Cx43 protein expression and PKC inhibitors prevented the increase in Cx43 expression induced by OxyHb. **a** The upper photographs are from a representative immunoblot shows Cx43 protein levels among different OxyHb incubation time points of experimental CVS model in cultured BASMCs, and quantitative graphs are shown below. Data represent the mean ± SEM, **P<0.01 vs. normal group, n = 8. **b** The effect of PKC inhibitors on Cx43 protein expression at the time of 48 h after OxyHb incubation. Quantitative graphs are shown below, data represent the mean ± SEM, *P<0.05, **P<0.01 vs. normal group, ^##^P<0.01 vs. OxyHb-only group, n = 8. The full-length blots are presented in Additional file 1: Figs. S1, S2

As shown in Fig. 1a of different groups, we observed that OxyHb (10^−6^ M) incubation in different time points, the Cx43 protein expression gradually increased with a maximal at 48 h, and then returned to control by 96 h. Exposure of the cells to CHE or GF alone had no effect on the level of Cx43 protein expression compared to normal cells, but both CHE and GF significantly prevented the maximal expression(48 h) in Cx43, suggesting a role of PKC in OxyHb-induced Cx43 expression (Fig. 1b).

### The effect of PKC inhibition on OxyHb-induced enhancement of gap junction intercellular communication in BASMCs

**Fig. 2:**
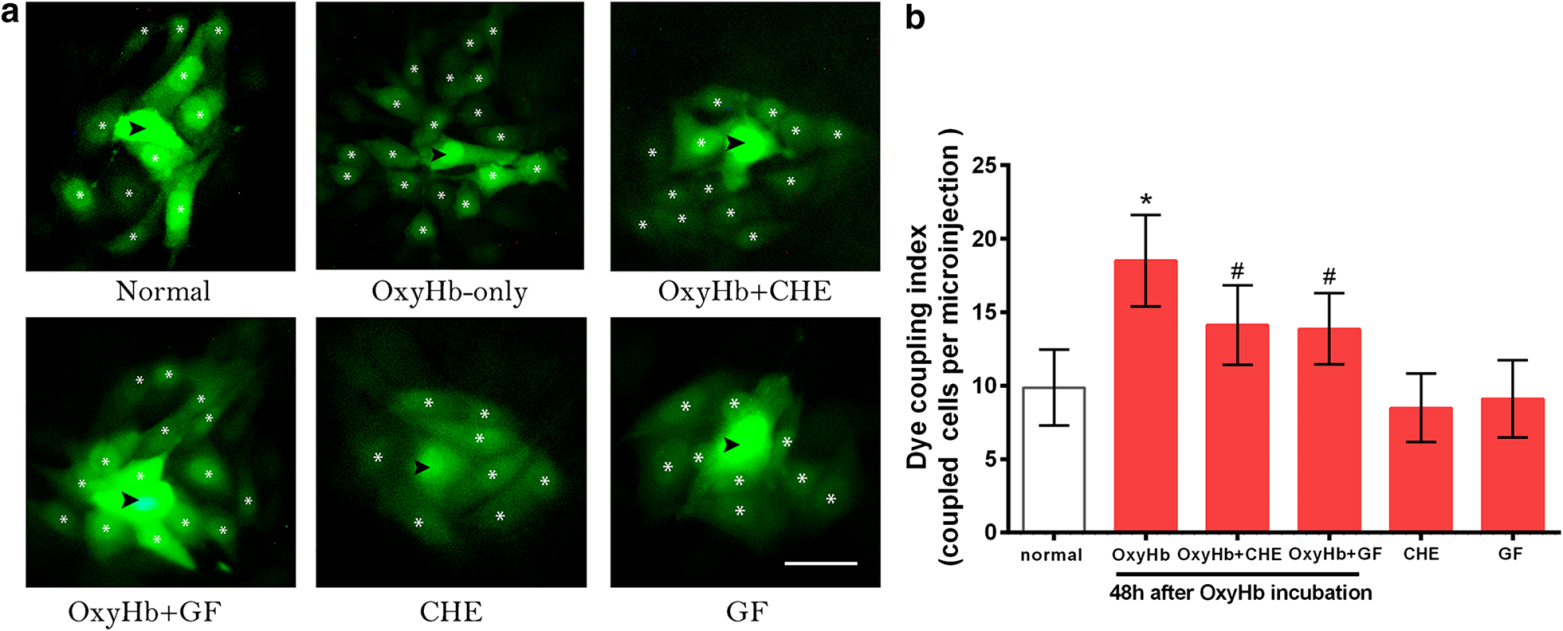
PKC inhibitors decrease the gap junction intercellular communication induced by OxyHb in BASMCs. **a** Representative photomicrographs show fluorescence images of one microinjected cell and its adjacent cells containing the dye tracer after injection of Lucifer yellow in each group. The injected cell is marked with a black arrow, neighboring cells which show dye transfer are marked with white asterisks; Scale bar = 5 μm. Under normal conditions 9.88 ± 2.59 cells were coupled, while in OxyHb-only group the number of coupled cells increased to 18.5 ± 3.12. After pre-treatment of CHE and GF, the coupled cells decreased significantly as 14.13 ± 2.70 and 13.88 ± 2.42. **b** Quantitative results of (**a**). Data represent the mean ± SEM, *P<0.01 vs. normal group, ^#^P<0.01 vs. OxyHb-only group, n = 8. The fluorescence images and corresponding brightfield images in each group are presented in Additional file 1: Fig. S3

Normally, an average of 9.88 ± 2.59 cells coupled to each cell was detected. Incubation with OxyHb for 48 h significantly increased the numbers of coupled cells to 18.5 ± 3.12 cells per microinjection (Fig. 2a, b, dye coupling index). As expected, both PKC inhibitors significantly reduced Lucifer yellow transfer by more than 20% (14.13 ± 2.70 and 13.88 ± 2.42, respectively, P < 0.05) when OxyHb was added with the pretreatment of CHE or GF. However, CHE or GF alone did not affect the migration of LY between cells compared with controls, which corresponded with our observation of Cx43 levels.

### In vivo experiment of the time-course changes in Cx43 protein levels among BAs

**Fig. 3:**
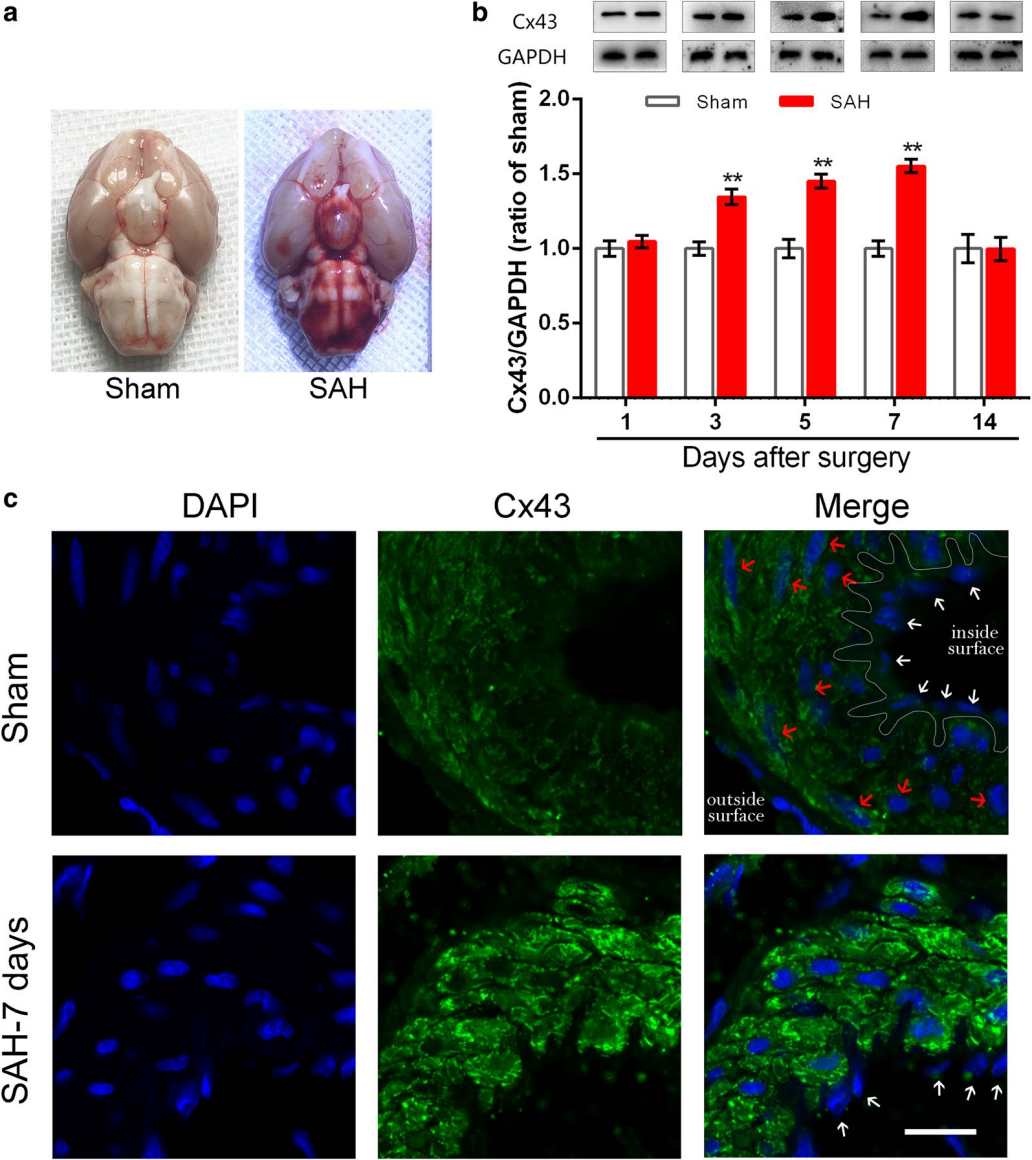
Immunolabeling of Cx43 in BAs derived from the sham and SAH groups. **a** Basal views of the brain in sham-operated and SAH animal. Blood was not observed in the sham group while blood are diffused in the subarachnoid space and around Willis’ circle in the SAH group; **b** the optic densities of Cx43 were normalized to that of sham rat at the corresponding time periods, SAH leads to increasing Cx43 expression in BAs. The upper photographs are from a representative immunoblot and quantitative graphs are shown below. Data represent the mean ± SEM, *P<0.05,**P<0.01, v.s. sham group at the corresponding time point, n = 8. The uncropped blots are presented in Additional file 1: Fig. S4. **c** Localization for DAPI and Cx43 in rat subjected to surgery. Tissues were taken on day 7 after surgery. White arrow: endothelium cell (EC); red arrow: smooth muscle cell (SMC); white line: internal elastic lamina. Scale bar = 15 μm. Images of day 1, 3, 5 and 14 are presented in Additional file 1: Fig. S7

In the SAH group, the blood was observed diffusely distributed in the subarachnoid space and around Willis’ circle on day 7 (Fig. 3a). In the sham group, no significant change in Cx43 was found throughout the time course after surgery. However, similar to the in vitro findings, Cx43 alterations in the BAs were also time dependent in the SAH group compared to the corresponding time periods in the sham group in vivo. Cx43 was significantly increased from the first day and continuously elevated to a peak value at 7 days following SAH; 14 days after SAH induction, the expression levels of Cx43 were similar in the sham and control SAH groups (Fig. 3b) which further verified our hypothesis. In addition, immunohistochemistry was performed to identify the cell type that expresses Cx43. In the sham-operated group (Fig. 3c, upper panel), Cx43 was colocalized with DAPI, and Cx43-stained punctate was abundant around the smooth muscle cells of all the BAs examined, suggesting pericellular localization. In the media, discrete spots were distributed circumferentially around the vessel wall, mimicking the orientation of the smooth muscle cells, which is consistent with previous findings [41]. However, beneath the internal elastic lamina, it was difficult to distinguish the staining around the ECs (Fig. 3c, white arrow). Staining was rarely observed around the ECs in the SAH group, similar with the sham-operated group. This finding suggests that in BAs, Cx43 is mainly expressed in the SMC layer rather than in the ECs. Consistent with the Western blotting findings, compared with the Cx43 immunofluorescence intensity of the sham-operated group, that of the SAH group increased gradually from 1 to 3 and 5 days after SAH and then peaked on day 7 (Fig. 3c, lower panel). Fourteen days after SAH, Cx43 immunofluorescence intensity had returned to normal levels (Additional file 1: Fig. S7). Hence, our hypothesis was further verified.

### Effects of Cx43 siRNA and PKC inhibitors on the experimental therapeutic group of rats

**Fig. 4:**
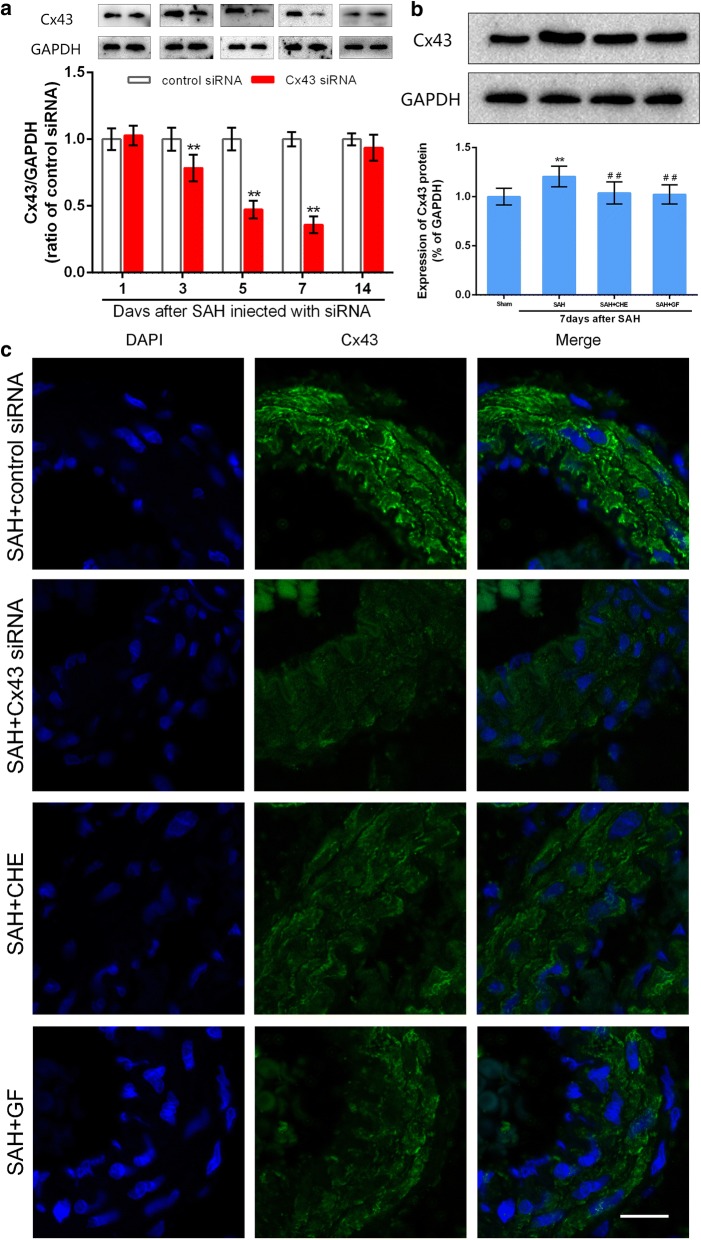
Pretreatment of Cx43-targeting siRNA leads to knockdown of BA Cx43 expression and PKC inhibitors reversed the increased Cx43 on day 7 after SAH. **a** After cisterna magna injection with either non-targeting siRNA (control) or Cx43-targeting siRNA, expression levels of Cx43 in BAs were assessed by Western blotting over time (days). The upper photographs are from a representative immunoblot and quantitative graphs are shown below. **P<0.01 vs. rat injected with control siRNA at the corresponding time point, n = 8. Bars indicate the mean ± SEM. The uncropped blots are presented in Additional file 1: Figs. S5, S6. **b** The effect of PKC inhibitors on Cx43 protein level on day 7 after SAH. Quantitative graphs are shown below, data represent the mean ± SEM, **P<0.01 vs. normal group, ^##^P<0.01 vs. OxyHb-only group, n = 8. **c** Localization for DAPI and Cx43 in rat subjected to surgery. Tissues were taken 7 days after SAH in each group. From top to bottom the groups are: SAH + control siRNA, SAH + Cx43 siRNA, SAH + CHE and SAH + GF, respectively. Scale bar = 15 μm

Cx43 expression gradually increased to day 7 and then decreased to normal levels by day 14 in the control siRNA group with SAH induction. In striking contrast, no significant elevation was detected along the same time course in the Cx43 siRNA group. In comparison with the levels at the corresponding time periods in the control siRNA group, the efficacy of Cx43 attenuation was observed beginning at day 3 after cisterna magna injection of Cx43-targeting siRNA. On day 3, 5 and day 7 in the Cx43 siRNA group, the expression of Cx43 tended to be significantly lower than that of the non-targeting siRNA group (Fig. 4a). In another experimental therapeutic group, the peak increased expression of Cx43 in the BAs at day 7 post-SAH was reversed by using either PKC inhibitor, CHE or GF (Fig. 4b).

Furthermore, immunohistochemistry revealed that Cx43 expression in the SMC layer was reduced 7 days after the injection of Cx43 siRNA compared with that of the non-targeting siRNA (Fig. 4c, upper panels). Immunohistochemistry also showed that Cx43 expression in the SMC layer was reduced in the two SAH + PKC inhibitor groups (Fig. 4c, lower panels) compared with that in the SAH-only group (Fig. 3c); this finding verified that PKC inhibitors reverse the increase in Cx43 expression.

### Knockdown of Cx43 via siRNA and PKC inhibitors leads to remission of vasospasm and reversal of CBF after SAH in rats

**Fig. 5:**
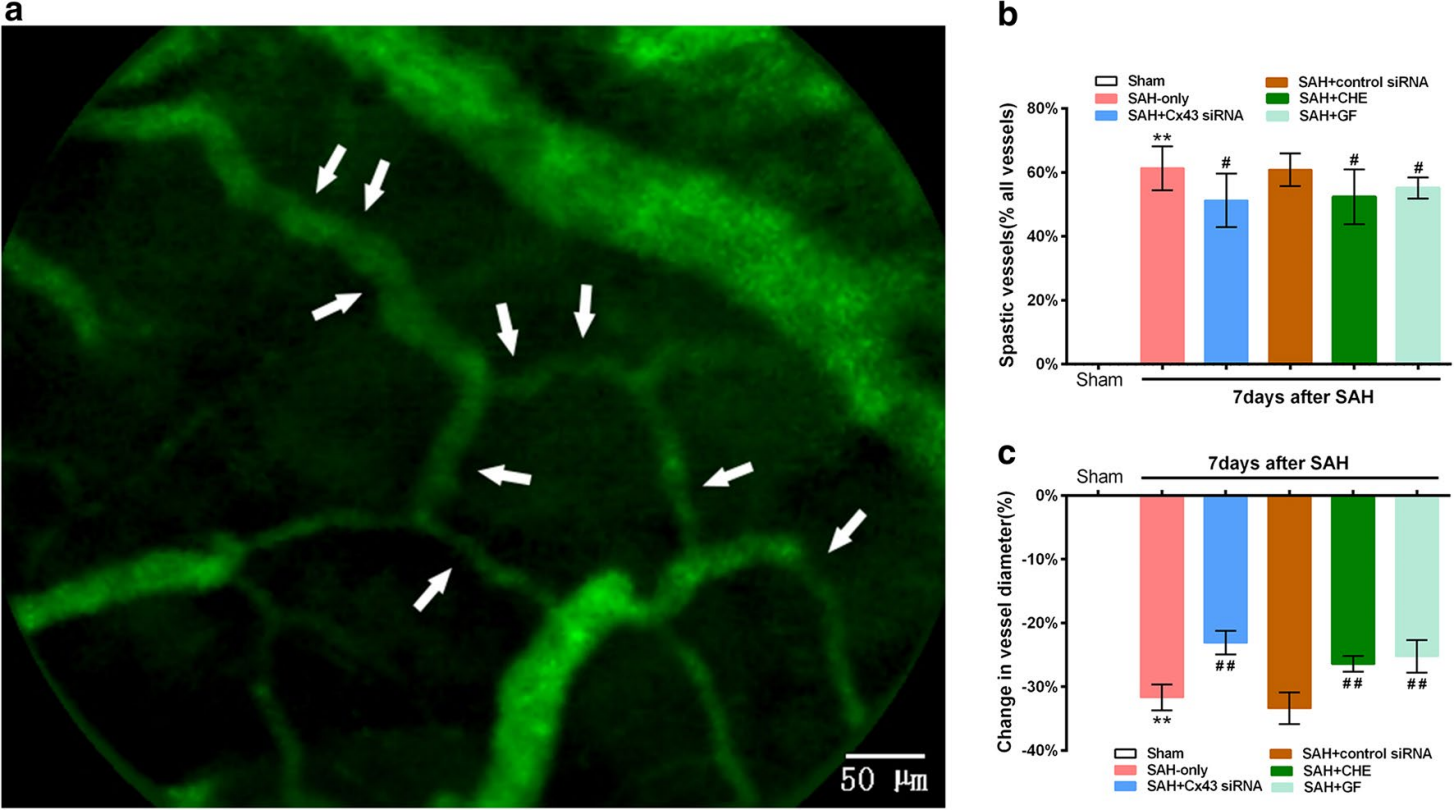
Cx43 siRNA and PKC inhibitors alleviated the changes in proportion and severity of microarterioles on SAH animals. **a** Examples of pearl string-like microarteriolar spasm (white arrow) in the cerebral microcirculation on 7 days after SAH. By investigating the morphology of microarteriolar spasm under intravital microscopy two sides of microarteriolar spasm represent the normal value of diameter (bar = 50 μm). **b** The proportion of spastic vessels quantified as percentage of all vessel segments among the ipsilateral MCA at 7 days after SAH. **c** The severity of vasospasm represented as change in diameter at 7 days after SAH (n = 8, **P<0.01 vs. sham group. ^#^P<0.05, ##P<0.01 vs. SAH-only group). Bars indicate the mean ± SEM

No microvascular constrictions were observed in sham-operated rats, and numerous constricted microvessels were observed at 7 days after SAH. As expected, the vasospastic pattern is similar to the pearl string-like constrictions observed in SAH patients. Quantification of these observations (Fig. 5b) revealed that, at 7 days after SAH, a significantly increased proportion (P < 0.01 vs. sham group, n = 8) of vessel segments derived from the MCA showed one or more constrictions in the SAH-only (61.33 ± 6.87%) and SAH + control siRNA (60.83 ± 5.12%) groups. Nevertheless, though an increased proportion (P < 0.01 vs. sham group, n = 8) of constricted vessel segments was also observed in the SAH + Cx43 siRNA group (51.28 ± 8.37%), knockdown of Cx43 via siRNA decreased the proportion of constricted vessel segments compared with the SAH-only group (P < 0.05). In parallel with the Western blot findings, the two SAH + PKC inhibitor groups also showed decreased proportions to different extents, suggesting the important role of Cx43 in microvascular spasms after SAH and the involvement of the PKC pathway. In addition, the severity of microvessel constriction was assessed by changes in microvessel diameters (Fig. 5c). Seven days after SAH, the diameters of affected vessel segments were significantly decreased in the SAH-only and SAH + control siRNA groups compared with the sham group (P < 0.01). In the SAH + Cx43 siRNA group and the two SAH + PKC inhibitor groups, the severity of constriction led to remission compared with the SAH-only group (P < 0.01). These data indicated increases in both the proportion and severity of microvasospasms at 7 days after SAH, which could be alleviated by knockdown of Cx43 via RNA interference and PKC inhibition.

**Fig. 6:**
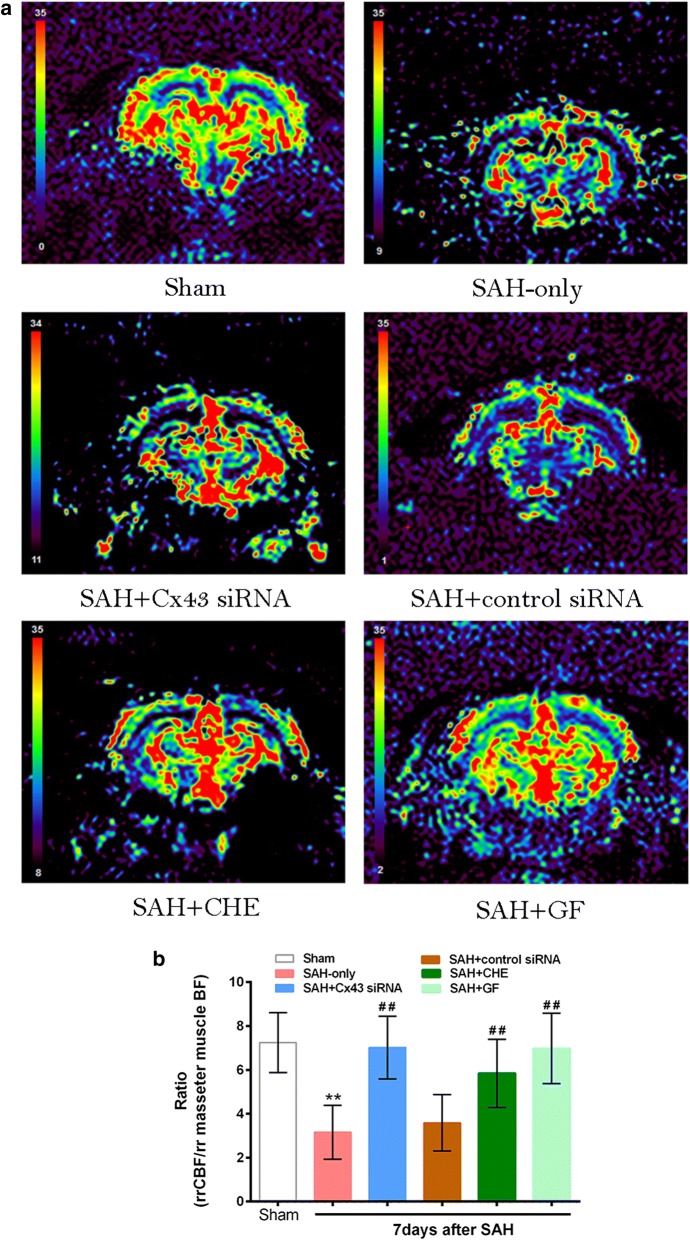
Cx43 siRNA and PKC inhibitors reversed the reduction of CBF on SAH animals. **a** Representative CBF color maps in hippocampus slice from anesthetized rats ventilated with room air in response to different experimental groups; **b** quantitative graph of ratio of regional relative CBF/regional relative masseter muscle BF. Blood flow of the masseter muscle was used as a reference, the rr CBF/rr masseter muscle BF ratio serve as a quantitative indicator for analyzing reduced CBF. By choosing hippocampus area and masseter muscle as regions of interest for the calculation of the defined hippocampus area/masseter muscle ratio, ratio reduced in the animal suffering from CVS whereas the ratio rose in therapeutic groups. **P<0.01 vs. sham group. ^##^P<0.01 vs. SAH-only group, n = 8. Bars indicate the mean ± SEM

rrCBF was measured via MR PWI, which is a general semiquantitative method, and the contralateral hemisphere was used as a reference [42–44]. However, in SAH-induced CVS, the reduction of CBF is not considered to be unilateral [45, 46]. So in the present study, blood flow of the masseter muscle was used as a reference [37]. According to previous studies, the CBF/masseter muscle BF ratio in male Sprague–Dawley rats was reported within a range of 6.1–7.7. Thus, the rr CBF/rr masseter muscle BF ratio may serve as a good quantitative indicator for analyzing reduced CBF. Here, rrCBF was 7.25 ± 1.37-fold higher compared to the perfusion of the rr masseter muscle in sham-operated rats, which is consistent with previous studies [47, 48]. However, the ratio was reduced to 3.16 ± 1.23 on day 7 after SAH. In our previous study [8] we demonstrated that Cx43 increased, and vasospasms occurred in SAH animals. Together with the observation above that Cx43 was significantly increased on day 7, our data demonstrate a significant correlation between the defined ratio (CBF/muscular BF) and the increased Cx43 expression 7 days after SAH with DCI. Additionally, on day 7 post-SAH, both the SAH + Cx43 siRNA and SAH + PKC inhibitor (CHE or GF) groups exhibited a significantly smaller reduction in CBF, while there was no significant change in the SAH + control siRNA group (Fig. 6).

## Discussion

Our present study showed that Cx43 protein was increased in the in vitro CVS model with OxyHb incubation. Moreover, our dye transfer assay also revealed enhanced GJIC in BASMCs in culture incubated with OxyHb. Our previous study demonstrated that Cx43 expression may up-regulate contractions in rabbit BAs via a GJIC-dependent mechanism evaluated by a scrape-loading method [49], we further confirmed here. The enhancement in GJIC may lead to exaggerated contractile responses among more coupled SMCs. Thus, spasmogenic signals such as red blood cell breakdown and the metabolic products spreading into the subarachnoid space could propagate more rapidly and forcefully from the ruptured vessel wall to a more extensive area, causing a prolonged contractile response. In addition, in our previous study, we showed that heptanol, a gap junction inhibitor, significantly inhibited the sustained contraction of rabbit BA rings induced by OxyHb in a dose-dependent manner [12]. The implication of gap junctions, especially Cx43, in OxyHb-induced CVS was further demonstrated here.

Both specific inhibitors of PKC we selected (CHE/GF) have been used to study the involvement of PKC in signal transduction pathways [27, 28]. By treatment with the two PKC inhibitors, the increased Cx43 protein and the enhanced GJIC were both reversed, implying that OxyHb-induced Cx43 alterations and enhanced GJIC may be modulated via the PKC pathway. However, previous studies have reported that PKC is composed of a family of serine–threonine kinases consisting of three major classes with at least 11 different isoforms [50]. During the pathological process of canine basilar artery cells in previous CVS research [24], PKCδ translocates from the cytosol to the membrane in early stage, and PKCα is subsequently translocated, suggesting that PKCδ plays a role during initiation and PKCα in the maintenance of CVS. However, as the two PKC inhibitors used here are both non-selective, the specific isoforms of PKC and their functional roles leading to Cx43 alterations are presently unknown and their identity remains to be elucidated. Furthermore, Richards et al. [51] proposed that PKC spatially and temporally controls GJIC via phosphorylation of Cx43 at serine 368, meanwhile as PKC activity was significantly enhanced in the membrane fraction of vasospastic cerebral arteries, the enhanced GJIC revealed here may be associated with the phosphorylation level of Cx43 at serine 368. Interestingly, given that the total expression of Cx43 protein also increased significantly in our observations, it seems that the modulation of GJIC may result from the ratio of pS368-Cx43 to total Cx43, which remains to be explored.

In our previous study [8], we found that vasospasm was most intense and coincidentally that the expression of Cx43 peaked on day 7. Together with our observation of the time phase change of Cx43 on SMCs, our findings further determined the involvement of increased Cx43 in SAH-induced CVS both in vivo and in vitro. On the other hand, DCI is thought to be caused by the combined effects of cortical spreading ischemia, arteriolar constriction and thrombosis. Here, DCI was investigated by performing intravital fluorescence microscopy and MRI measurements by PWI in rats. We directly visualized the post-SAH microcirculation in vivo by intravital fluorescence microscopy. This technique is advantageous over ex vivo studies for investigating arterioles in the living brain, allowing us to observe vasospasms and dynamic interactions in the entire vascular tree of MCA. Our main findings of the DCI investigation were that CBF significantly decreased and over 60% of arterioles derived from the MCA showed pearl string-like constrictions on day 7 after SAH. As reducing vessel diameter by 30% could reduce flow by ~ 80%, leading to DCI, our results may explain why DCI is observed after SAH in both experimental animals and patients [51–53]. It suggesting that changes in Cx43 might be implicated in the pathophysiological events of DCI since both DCI and increment of Cx43 coincidentally occurred on day 7.

After knockdown of Cx43 via RNA interference, MCA deficiency and reduced CBF were both eased while control siRNA did not significantly relieve the deficits. In a previous study [54], corrosion casts of arterioles showed that they exhibited tapered narrowing with folding after SAH. Additionally, the width of the arterioles was significantly lower at 3 and 7 days after SAH. Morphometric examination by light microscopy showed that the internal diameter of the arterioles was significantly smaller and that this change was associated with a significant increase in wall thickness at any depth from the brain at 7 days after SAH. This condition had improved by 14 days after SAH, suggesting that arteriole constriction occurs after SAH and may contribute to DCI. A previous study [55] used orthogonal polarization spectral (OPS) imaging and found that in SAH patients, capillary density was significantly lower and the small arteries and arterioles at the cortical surface exhibited vasospasm that was not detected by angiography or transcranial Doppler sonography. These changes indicated that CVS may be associated with a significant decrease in capillary perfusion. Although Cx43 expression was restored by pretreatment with PKC inhibitors or Cx43 siRNA, resulting in the significant alleviation of the deleterious effects of SAH, the microvascular spasms were not completely reversed (Fig. 5). In our Cx43 siRNA interference experiment (Fig. 4a), the efficacy reached an ideal level prior to days 3–5. Because the development of constriction in microarterioles is a gradual process and because a previous study in rats [56] showed that subarachnoid arterioles constrict by ~ 40% in the early phase, i.e., 5 min to 2 h after SAH in vivo, we suspect that the efficacy of Cx43 siRNA interference and the occurrence of micro-constriction are not synchronously correlated. Thus, in accordance with our proposed hypothesis, we showed that the severity of CVS and, subsequently, DCI induced by SAH were strongly related to vascular Cx43 alterations. Moreover, in addition to SMC, astrocytes are also important regulators of vasomotor reaction. The high abundance of gap junctions in astrocytes allows for the direct intercellular diffusion of ions, nutrients, and signaling molecules between these cells. Cx43 is the most abundant connexin expressed in astrocytes and thus constitutes the major connexin contributing to gap junction communication in astrocytes. Hence, SAH-induced alterations in the expression and functions of Cx43 should be systematically explored in other types of cells in future studies.

Endothelium-mediated vasodilatation is evoked by increased calcium concentrations in the endothelium, which triggers hyperpolarization by activating endothelial calcium-dependent potassium channels [57], leading to the recognition of endothelium-dependent hyperpolarizing factor (known as EDHF). Thus, the conduction of EDHF via MEGJs to the underlying smooth muscle cell layer results in a decrease in intracellular calcium and subsequent vasodilatation. Because vasodilation signals upon focal stimulation of arterioles have been shown to be conducted along the vascular wall [58], Hoepfl B et al. [59] suggested that EDHF, but not NO or prostaglandins, serves as a critical mediator to initiate conducted vasodilation (CVD) upon acetylcholine administration in hamster arterioles. In the present study, Cx43 increased significantly after SAH, and it is believed that progressive up-regulation of Cx43 in SMCs may result in the development of intimal hyperplasia [60], and increasing intimal hyperplasia mechanically dissociates endothelial–medial interactions at the MEGJ interface. Decreasing myoendothelial interactions progressively attenuates the hyperpolarization that normally passes from the ECs to medial SMCs, eventually leading to irregular vasomotion. Moreover, studies using connexin-mimetic peptides to selectively inhibit GJIC in rabbit iliac arteries suggested that Cx43 is required for propagation of EDHF within the smooth muscle layer [61], implying the involvement of Cx43 in disruption of CVD. Although our data demonstrated that Cx43 is closely related to the CVS in pathological conditions of SAH, these findings do not explain the specific mechanism of Cx43 underlying the impaired vasodilation. Although SMCs have been reported to primarily express Cx43 [41] and ECs to primarily express Cx40 [62, 63], the connexin content of MEGJs remains unknown and has not been adequately explored. In contrast with Cx43 knockout mice, constitutive deletion of Cx40 results in hypertension in both anesthetized and awake mice [64, 65]. Cx40 is also critical for transferring vasodilatory signals from ECs to the vascular media [66]. Moreover, it has been proposed that nearly a twofold difference in the Cx43:Cx40 expression ratio gives rise to more than a 25-fold difference in dye coupling via the GJIC in vitro [67]. Together with our observation that Cx43/Cx40 proteins form heteromeric gap junctions that are increased in SAH, we hypothesize that the Cx43:Cx40 ratio maintains a certain pattern for the maintenance of various physiological functions under normal conditions. Once SAH occurred, the connexin patterns varied, and both increased Cx43 and decreased Cx40 within the cerebral vessel wall may be responsible for the development of CVS. The present findings suggest a novel emphasis on this research direction for future studies.

## Conclusion

In summary, the important role of Cx43 in SAH-induced CVS was affirmed in present study. Gap junction remodeling occurred after SAH and may be implicated in irregular vasomotion of CVS. Moreover, increased Cx43 after SAH may be modulated via the PKC pathway.

## Supplementary information

**Additional file 1: Fig. S1.** Full length blots of Fig. 1A, which include western blotting analysis of Cx43 expression time-course change after OxyHb incubation in vivo. The numbers represent different treatment groups: 1: normal; 2: 24h; 3: 48h; 4: 72h; 5: 96h. **Fig. S2.** Full length blots of Fig. 1B: Western blotting analysis of the effect of PKC inhibitors on Cx43 protein expression at the time of 48h after OxyHb incubation. The numbers represent different treatment groups: 1: normal; 2: OxyHb-only; 3: OxyHb+CHE; 4: OxyHb+GF; 5: CHE-only; 6: GF-only. **Fig. S3.** Fluorescence images and corresponding brightfield images in each group. The injected cell is marked with a black arrow. Scale bar = 50 μm. **Fig. S4.** Blot images of Fig. 3B: Western blotting analysis of Cx43 in BAs derived from the sham and SAH groups. The numbers represent different treatment groups: 1: sham; 2: SAH. **Fig. S5.** Blot images of Fig. 4A: Western blotting analysis of Cx43 in BAs derived from the non-targeting siRNA (control) or Cx43-targeting siRNA groups after SAH. The numbers represent different treatment groups: 1: control siRNA; 2: Cx43 siRNA. **Fig. S6.** Blot images of Fig. 4B: Western blotting analysis of Cx43 in BAs derived from the 2 PKC inhibitors groups after SAH. The numbers represent different treatment groups: 1: sham; 2: SAH-only; 3: SAH+CHE; 4: SAH+GF. **Fig. S7.** Immunolocalization for DAPI and Cx43 in rat subjected to surgery. Tissues were taken 1,3,5 and 14 days after SAH in each group. Scale bar = 5 μm.

## Data Availability

All data and materials supporting the conclusion were included in this main paper.

## Abbreviations

BAs: basilar arteries
BASMCs: basilar arterial smooth muscle cells
CHE: chelerythrine
CVS: cerebral vasospasm
Cx43: connexin 43
DCI: delayed cerebral ischemia
ECs: endothelium cells
EDHF: endothelium-derived hyperpolarizing factor
GF: GF 109203X
GJIC: gap junction intercellular communication
HVJ-E: HVJ envelope
LY: lucifer yellow
MCA: middle cerebral artery
MEGJ: myo-endothelial gap junction
MR PWI: magnet resonance-perfusion weighted imaging
OxyHb: oxyhemoglobin
PKC: protein kinase C
ROIs: regions of interests
rr CBF: relative regional cerebral blood flow
SAH: subarachnoid hemorrhage
SMCs: smooth muscle cells
