# Supplementary material for: The important role of connexin 43 in subarachnoid hemorrhage-induced cerebral vasospasm
Source: J Transl Med. 2019 Dec 30;17:433. doi: 10.1186/s12967-019-02190-1 (PMC6936071; doi:10.1186/s12967-019-02190-1)
Supplement: Supplementary file 1 — Additional file 1: Fig. S1. Full length blots of Fig. 1A, which include western blotting analysis of Cx43 expression time-course change after OxyHb incubation in vivo. The numbers represent different treatment groups: 1: normal; 2: 24h; 3: 48h; 4: 72h; 5: 96h. Fig. S2. Full length blots of Fig. 1B: Western blotting analysis of the effect of PKC inhibitors on Cx43 protein expression at the time of 48h after OxyHb incubation. The numbers represent different treatment groups: 1: normal; 2: OxyHb-only; 3: OxyHb+CHE; 4: OxyHb+GF; 5: CHE-only; 6: GF-only. Fig. S3. Fluorescence images and corresponding brightfield images in each group. The injected cell is marked with a black arrow. Scale bar = 50 μm. Fig. S4. Blot images of Fig. 3B: Western blotting analysis of Cx43 in BAs derived from the sham and SAH groups. The numbers represent different treatment groups: 1: sham; 2: SAH. Fig. S5. Blot images of Fig. 4A: Western blotting analysis of Cx43 in BAs derived from the non-targeting siRNA (control) or Cx43-targeting siRNA groups after SAH. The numbers represent different treatment groups: 1: control siRNA; 2: Cx43 siRNA. Fig. S6. Blot images of Fig. 4B: Western blotting analysis of Cx43 in BAs derived from the 2 PKC inhibitors groups after SAH. The numbers represent different treatment groups: 1: sham; 2: SAH-only; 3: SAH+CHE; 4: SAH+GF. Fig. S7. Immunolocalization for DAPI and Cx43 in rat subjected to surgery. Tissues were taken 1,3,5 and 14 days after SAH in each group. Scale bar = 5 μm. [file 12967_2019_2190_MOESM1_ESM.doc]

**The important role of connexin 43 in subarachnoid hemorrhage-induced cerebral vasospasm**

**Le Yang** **^+^, Jian Yan** **^+^, Jin An Zhang, Xin Hui Zhou, Chao Fang, Er Ming Zeng, Bin Tang, Jian Duan, Guo Hui Lu and Tao Hong***

Department of Neurosurgery, the First Affiliated Hospital Of Nanchang University, 17 Yong Wai Zheng Street, Nanchang 330006, People’s Republic of China

*Corresponding author. E-mail: ht2000@vip.sina.com

**^+^** These authors contributed equally to this work.

**Additional file 1**

**
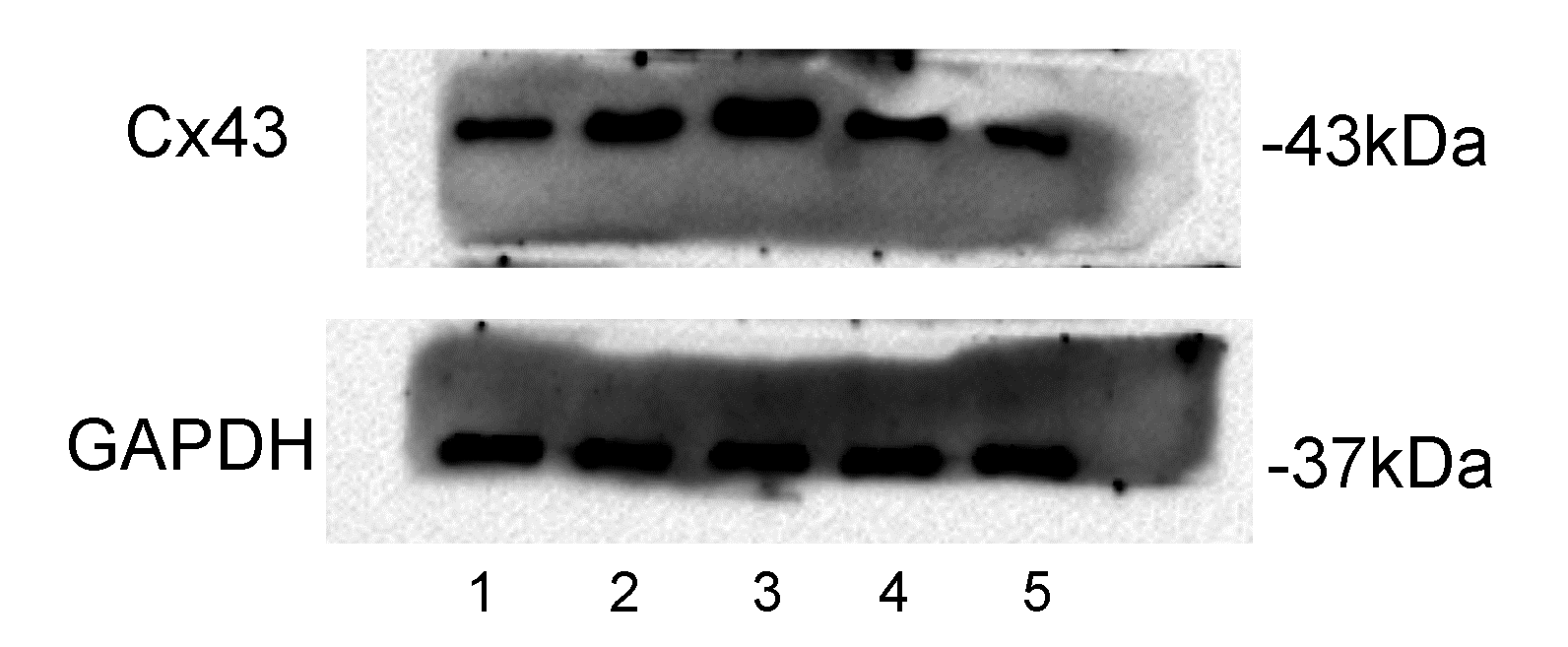
**

Fig.S1：Full length blots of Fig.1A, which include western blotting analysis of Cx43 expression time-course change after OxyHb incubation in vivo. The numbers represent different treatment groups: 1: normal; 2: 24h; 3: 48h; 4: 72h; 5: 96h.

**
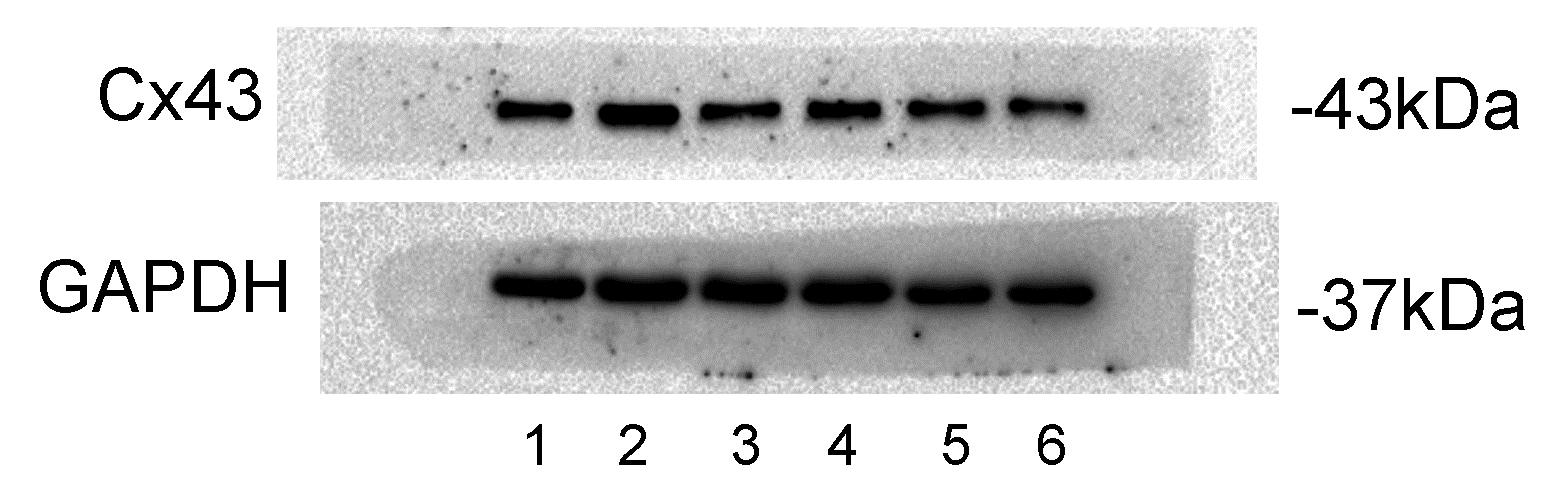
**

Fig.S2：Full length blots of Fig.1B: Western blotting analysis of the effect of PKC inhibitors on Cx43 protein expression at the time of 48h after OxyHb incubation. The numbers represent different treatment groups: 1: normal; 2: OxyHb-only; 3: OxyHb+CHE; 4: OxyHb+GF; 5: CHE-only; 6: GF-only.

**
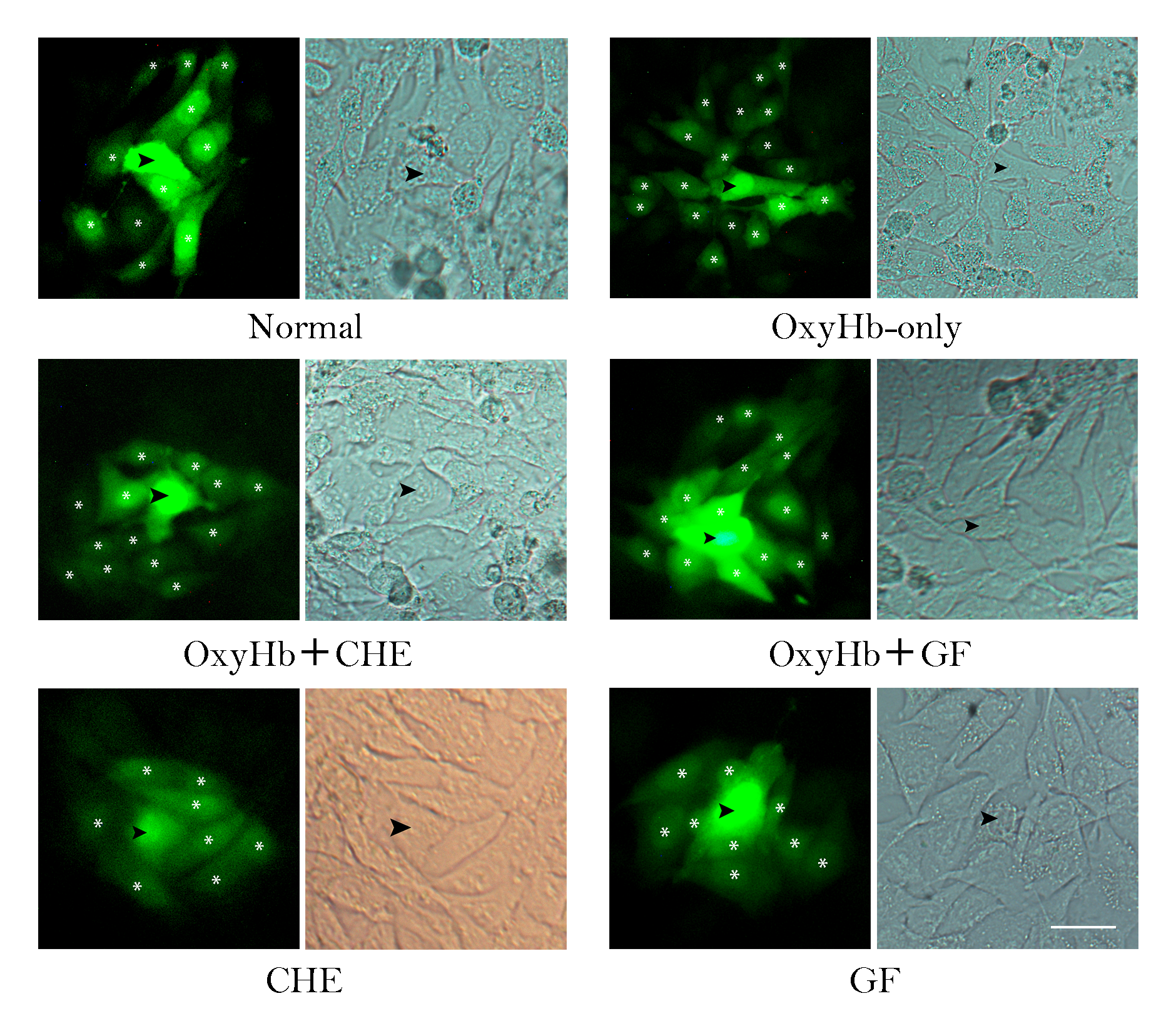
**

Fig.S3: Fluorescence images and corresponding brightfield images in each group. The injected cell is marked with a black arrow. Scale bar = 50 μm.


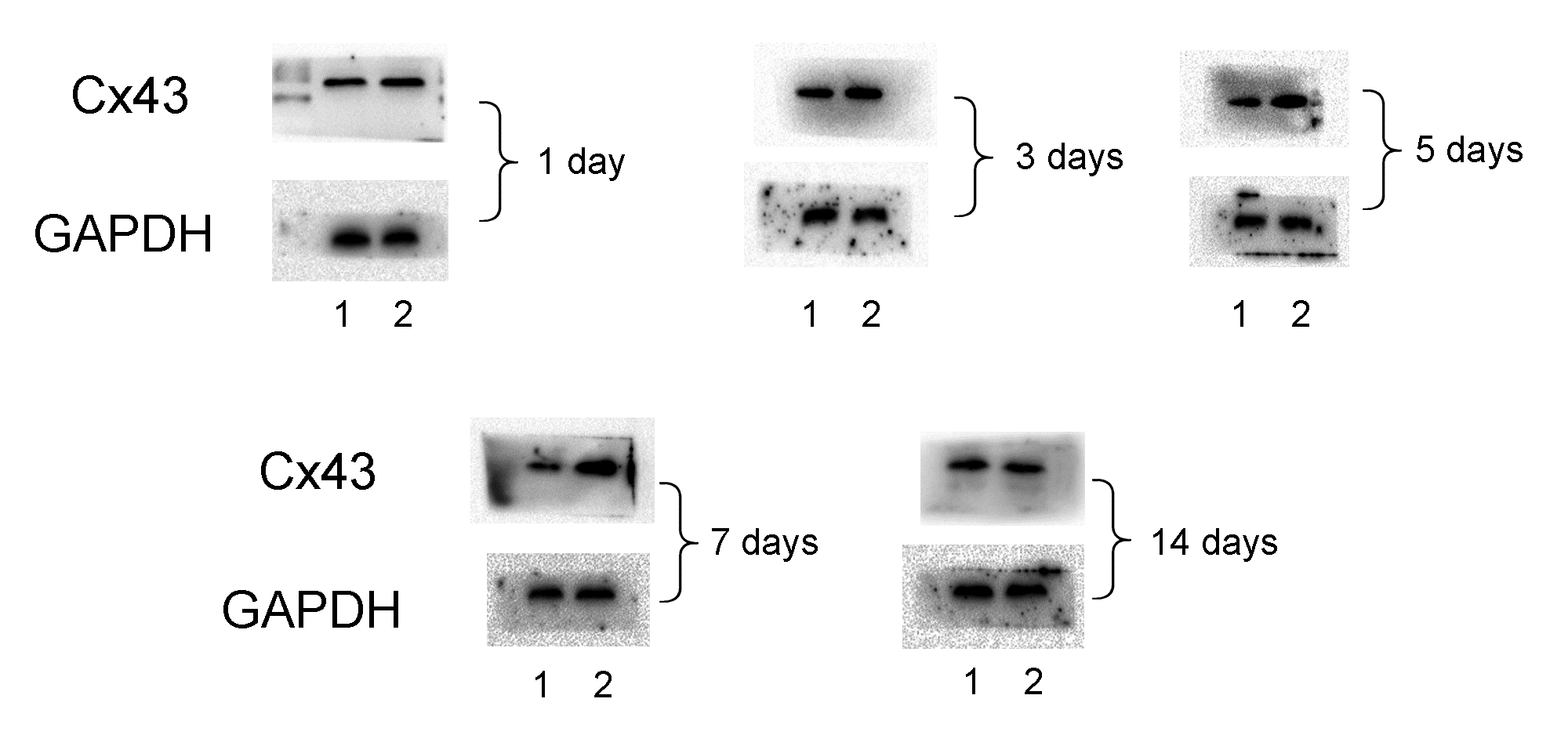


Fig.S4：Blot images of Fig.3B: Western blotting analysis of Cx43 in BAs derived from the sham and SAH groups. The numbers represent different treatment groups: 1: sham; 2: SAH.


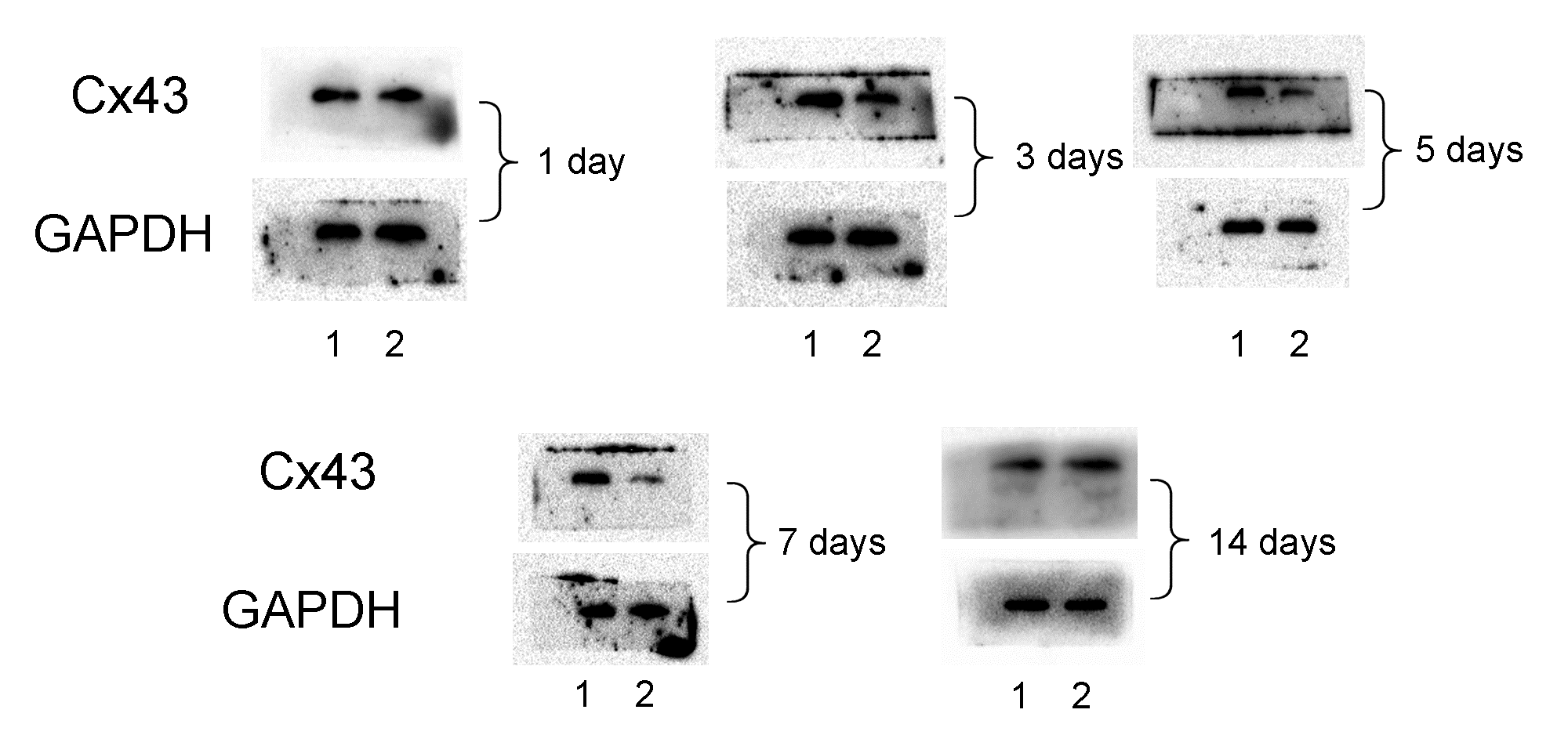


Fig.S5：Blot images of Fig.4A: Western blotting analysis of Cx43 in BAs derived from the non-targeting siRNA (control) or Cx43-targeting siRNA groups after SAH. The numbers represent different treatment groups: 1: control siRNA; 2: Cx43 siRNA.

**
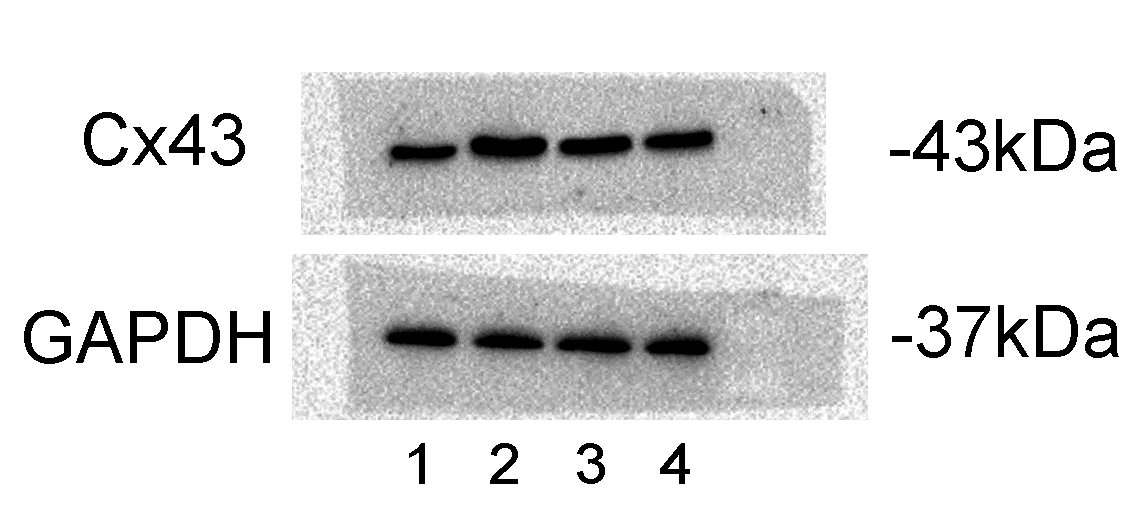
**

Fig.S6：Blot images of Fig.4B: Western blotting analysis of Cx43 in BAs derived from the 2 PKC inhibitors groups after SAH. The numbers represent different treatment groups: 1: sham; 2: SAH-only; 3: SAH+CHE; 4: SAH+GF.


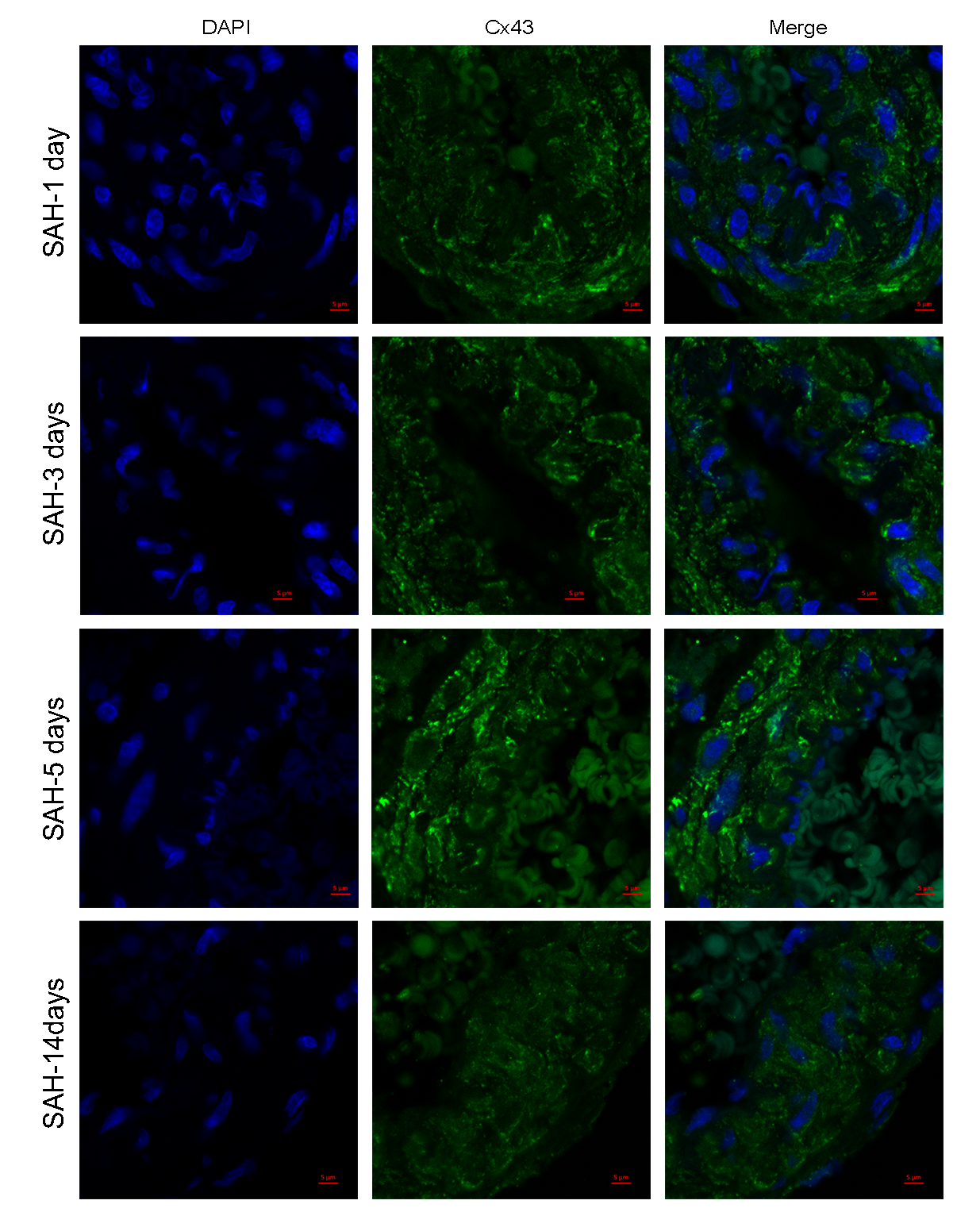


Fig.S7：Immunolocalization for DAPI and Cx43 in rat subjected to surgery. Tissues were taken 1，3，5and 14 days after SAH in each group. Scale bar = 5 μm.
